# Gut microbiota bidirectionally influences protection and severity in cerebral malaria in mice

**DOI:** 10.1186/s41182-026-00947-1

**Published:** 2026-04-16

**Authors:** Tomoyo Taniguchi, Eiji Miyauchi, Reika Kawabata-Iwakawa, Masahiko Nishiyama, Rika Umemiya-Shirafuji, Hiromu Toma, Nobuo Sasaki, Hajime Hisaeda, Haruyoshi Tomita, Hiroshi Ohno, Hidehiro Kishimoto, Hiroshi Suzuki

**Affiliations:** 1 Department of Immunology and Parasitology, Graduate School of Medicine, University of the Ryukyus, 1076 Kiyuna, Ginowan, Okinawa 901-2720 Japan; 2 National Research Center for Protozoan Diseases, Obihiro University of Agriculture and Veterinary Medicine, Nishi 2 Sen-13, Inada-Cho, Obihiro, Hokkaido 080-8555 Japan; 3 Department of Parasitology, Graduate School of Medicine, Gunma University, 3-39-22 Showa Machi, Maebashi, Gunma 371-8511 Japan; 4 Center for Medical Education, Graduate School of Medicine, Gunma University, 3-39-22 Showa Machi, Maebashi, Gunma 371-8511 Japan; 5 Laboratory for Intestinal Ecosystem, RIKEN Center for Integrative Medical Sciences, 1-7-22 Suehiro, Tsurumi, Yokohama, Kanagawa 230-0045 Japan; 6 Institute for Molecular and Cellular Regulation, Gunma University, 3-39-22 Showa Machi, Maebashi, Gunma 371-8511 Japan; 7 Division of Integrated Oncology Research, Gunma University Initiative for Advanced Research, Gunma University, 3-39-22 Showa Machi, Maebashi, Gunma 371-8511 Japan; 8 Department of Parasitology, National Institute of Infectious Diseases (NIID), Japan Institute for Health Security (JIHS), Tokyo, 162-0052 Japan; 9 Department of Bacteriology and Laboratory of Bacterial Drug Resistance, Graduate School of Medicine, Gunma University, Maebashi, Gunma 371-8511 Japan

**Keywords:** *Plasmodium berghei* ANKA, Experimental cerebral malaria, Gut microbiota, Antibiotics, Gnotobiotic experiment

## Abstract

**Background:**

Malaria caused by *Plasmodium* parasites leads to severe complications, such as cerebral malaria; however, the influence of the gut microbiota on the pathogenesis of cerebral malaria remains unclear. Here, the effects of antibiotic-induced microbiota alteration on experimental cerebral malaria (ECM) were examined.

**Methods:**

Male C57BL/6N mice that were administered drinking water containing a four-antibiotic cocktail, ampicillin, neomycin, metronidazole and vancomycin, from 2 weeks before *P. berghei* ANKA (PbA) infection were used for the experiments. Disease progression, blood–brain barrier (BBB) integrity, immune responses, and gut microbiota composition were evaluated.

**Results:**

Approximately 80% of mice with modified gut microbiota avoided ECM and showed reduced BBB disruption and lymphocyte infiltration into the brain. 16S rRNA gene sequencing revealed specific bacterial species that contributed to either the protection or pathogenesis of ECM. The mono-colonization of germ-free mice revealed that distinct bacterial species could attenuate or exacerbate ECM symptoms, independent of antibiotic effects.

**Conclusions:**

The findings highlight that distinct gut microbial populations can modulate host susceptibility or resistance to ECM, underscoring the important influence of the intestinal microbiota on infectious disease outcomes. This work broadens the understanding of host‒microbe interactions and may inform new strategies for managing cerebral malaria through microbiota modulation.

**Supplementary Information:**

The online version contains supplementary material available at 10.1186/s41182-026-00947-1.

## Background

Malaria caused by *Anopheles* mosquitoes infected with *Plasmodium* parasites was estimated to cause 282 million cases of malaria and 610,000 deaths worldwide in 2024, with increases observed throughout the COVID-19 pandemic [1]. Cerebral malaria is the most severe complication of *P. falciparum* infection. Despite several decades of malaria vaccine development, only 2 vaccines, RTS, S and R21/Matrix-M [2], have been approved by the WHO, and many other barriers exist for malaria eradication [1]. To address this issue, we need new strategies, including the development of malaria vaccines and other approaches and tools.

Controlling the gut microbiota could be a new approach to treatment of malaria infection, during which gastrointestinal symptoms such as abdominal pain, vomiting and diarrhea are frequently observed [3–7]. Recently, advances have been made in understanding the effects of the gut microbiota on host health and disease [8, 9], including during malaria infection have been obtained. Since the first report of the association of gut bacteria with malaria infection [10], several studies have described that changes in the gut microbiota are associated with malaria infection [11–17]. Gut bacteria have been implicated in regulating the splenic germinal center response [13], metabolism [14], and immune memory [15]. *Lactobacillus* and *Bifidobacterium* treated mice have been shown to reduce *P. yoelii* 17XNL (PyNL) load [16], and certain *Bacteroides* bacteria have been shown to be causally associated with the risk of severe malaria [17]. In an experimental cerebral malaria (ECM) model, C57BL/6N (B6) mice infected with *P. berghei* ANKA (PbA) have been demonstrated to suffer from intestinal pathology associated with marked changes in the microbiota [11]. However, the association between the gut microbiota and the pathogenesis of malaria, especially cerebral malaria, is poorly understood.

In this study, the effects of the gut microbiota on the pathogenesis of ECM in B6 mice in which the gut microbiota was modified by antibiotics were examined.

## Methods

### Mice and parasites

Four- to twelve-week-old male C57BL/6NCrSlc mice were purchased from Japan SLC, Inc., and maintained under specific pathogen-free conditions. Four- to twelve-week-old male germ-free Tsl:C57BL/6NCr mice were purchased from Sankyo Labo Service Corporation, Inc., and maintained in flexible film isolator units for gnotobiotic experiments. Age- and sex-matched groups were used for the experiments.

PbA was provided by Nagasaki University with support in part by the National BioResource Project (NBRP), AMED, Japan. Blood-stage parasites for experimental infections were obtained from donor mice 5 days after inoculation with frozen stock. The mice were given drinking water containing four antibiotics (4AB), 1 g/L ampicillin, 1 g/L neomycin, 1 g/L metronidazole and 0.5 g/L vancomycin (FUJIFILM Wako Pure Chemical Corporation) beginning 2 weeks before infection and during infection. Mice were infected with 1 × 10^5^ parasitized red blood cells via intraperitoneal injection. Following infection, survival and parasitemia were monitored throughout the observation period. Parasitemia was assessed by determining the percentage of parasitized red blood cells in Giemsa-stained thin smears using blood from the tail vein under a microscope.

### Bacteria

To isolate both the amplicon sequence variants (ASV) 1 and ASV37 bacteria, the stool from naive or 4AB-treated C57BL/6 mice was homogenized and diluted with PBS. The dilutions were plated on AccuDia^™^ DHL Agar and AccuDia^™^ Heart Infusion Agar (Shimadzu Diagnostics Corporation) and incubated anaerobically with an AnaeroPack^™^ (Mitsubishi gas chemical company, Inc.) at 37 °C for 24 h. To isolate the ASV37 bacterium, we used BD Difco™ Lactobacilli MRS Agar plates with 0.5% calcium carbonate and incubated them anaerobically with an AnaeroPack^™^ at 37 °C for 2 days. The 16S rRNA genes from individual colonies were amplified using the 27F and 1492R primers, and fragments, including the V4 region, were sequenced using an Applied Biosystems 3130xl Genetic Analyzer (Thermo Fisher Scientific Inc.) to determine the colonies corresponding to ASV1 and ASV37. The isolated bacteria were subsequently grown in FastGene^™^ LB Broth Stick and Difco^™^ Lactobacilli MRS Broth and stored in 30–50% glycerol at − 80 °C.

### Gnotobiotic mice

Male germ-free C57BL/6 mice at 5–7 weeks of age were gavaged with ASV1 or ASV37 bacteria 3 times (ASV1: 1.2 × 10^9^ cfu/mouse/time; ASV37: 2.1 × 10^9^ cfu/mouse/time) after acclimatization for 1 week. All gnotobiotic mice were kept in isolators for 2–4 weeks before being used for experiments. The colonization of both strains in germ-free mice was confirmed (ASV1: 2.9 × 10^9^ cfu/g of stool; ASV37: 2.1 × 10^8^ cfu/g of stool), and no contamination was detected by the ICLAS Monitoring Center.

### Assessment of ECM and blood‒brain barrier function

Blood–brain barrier (BBB) function was assessed using Evans blue (EB) dye as described previously [11]. Briefly, the mice were intravenously injected with 2% EB (0.2 ml; FUJIFILM Wako Pure Chemical Corporation) and sacrificed and perfused with heparinized PBS 1 h later. The brains were surgically removed, weighed and placed in 100% formamide (2 ml; FUJIFILM Wako Pure Chemical Corporation) for 48 h at 37 °C to extract the EB dye. The absorbance was measured at 630 nm using an MTP-450Lab microplate reader (Corona Electric Co., Ltd.). EB concentrations were measured on the basis of a standard curve, and the results were adjusted to the weight of the dye per g of brain tissue.

### Assessment of pathological changes in the brains

Whole brains were excised from AB-treated and uninfected control mice or 7 days post-infection after perfusion and were fixed in 10% phosphate-buffered formalin. The brains were embedded in paraffin, and the sections (3 μm) of the brains were stained with hematoxylin and eosin (H&E) by Biopathology Institute Co., Ltd. The specimens were observed using a BIOREVO BZ-9000 microscope (Keyence corporation).

### Cell preparation

Mononuclear cells (MNCs) from the brain and spleen were isolated as previously described [18–20]. Briefly, the brain and spleen were removed after perfusion using PBS until the effluent was clear. Freshly harvested brains were placed on top of a cell strainer (100-μm mesh size; Greiner Bio-One Co., Ltd.) in a 50-mL tube, and the brain tissue was cut into small pieces. The brain was pressed through a cell strainer using a plunger and suspended in AccuDia^™^ Eagle’s MEM (Shimadzu Diagnostics Corporation) supplemented with 5 mM HEPES and 2% heat-inactivated newborn calf serum (Gibco). After centrifugation at 250 × g for 10 min at 4 °C, the pellet was dissolved in medium containing 0.1% Collagenase D (Roche Diagnostics K.K.) and 2 KU/mL Deoxyribonuclease I (Worthington Biochemical Corp), and mixtured by using rotator for 30 min at room temperature and then incubated on ice for 5 min to remove cell debris. After centrifugation at 250 × g for 10 min at 4 °C, the pellet was dissolved in 30% Percoll solution (Cytiva Global Life Sciences Technologies Japan), mixed, placed into 70% Percoll solution and centrifuged at 500 × g with no brakes for 20 min at room temperature. The interface was transferred to a new tube, washed once with medium and centrifuged at 500 × g for 10 min at 4 °C. The pellet was then resuspended in medium. Splenocytes were obtained by forcing the spleen through a 200-gauge stainless steel mesh. Splenocytes were used after erythrocyte lysis.

### Immunofluorescence test

The surface phenotypes of lymphocytes were identified by five- or six-color immunofluorescence tests [19, 21]. Fluoroscein isothiocyanate (FITC)-, phycoerythrin (PE)-, PE-Cy7-, PerCP-Cy5.5-, BV421-, or BV510-conjugated monoclonal antibodies (mAbs) were used. The mAbs used here were anti-CD3 (145-2C11), anti-CD4 (GK1.5), anti-CD8α (53-6.7) and anti-CD45 (30-F11) mAbs (BioLegend, TONBO biosciences). To prevent nonspecific binding of the mAbs, we added CD16/32 (2.4G2; TONBO biosciences) before staining with the labeled mAb. After staining and fixation, the cells were analyzed by a CytoFLEX S (Beckman Coulter).

### DNA extraction from stool samples

Stool samples collected from mice were immediately placed in RNA*later* (Thermo Fisher Scientific Inc., CA, USA) and stored at − 80 °C until use. Bacterial genomic DNA was isolated as described previously with modifications [11, 22, 23]. The bacterial pellet was suspended and incubated with lysozyme (15 mg/ml; FUJIFILM Wako Pure Chemical Corporation) at 37 °C for 1 h in 10 mM Tris–HCl/10 mM EDTA. Purified achromopeptidase (FUJIFILM Wako Pure Chemical Corporation) was added at a final concentration of 2,000 U/ml and then incubated at 37 °C for 30 min. The suspension was treated with 1% (wt/vol) sodium dodecyl sulfate (FUJIFILM Wako Pure Chemical Corporation) and proteinase K (1 mg/ml, Merck Japan, Tokyo, Japan) and incubated at 50 °C for 1 h. The lysate was treated with phenol/chloroform/isoamyl alcohol, and the DNA in the aqueous phase was precipitated by the addition of ethanol and pelleted via centrifugation at 5,000 × g at 4 °C for 15 min. The DNA pellet was rinsed with 70% ethanol, dried and dissolved in 1 × TE. DNA samples were purified by treatment with Ribonuclease Solution (10 mg/ml; NIPPON GENE CO., LTD.) at 37 °C for 30 min and precipitated by the addition of equal volumes of a 10% polyethylene glycol solution (PEG6,000-2.5 M NaCl). The DNA was pelleted via centrifugation at 18,000 × g at 4 °C for 10 min, rinsed with 75% ethanol and dissolved in 1 × TE.

### 16S rRNA gene sequencing

The V4 variable region of the 16S ribosomal RNA (rRNA) gene was amplified by PCR using dual barcoded primers as described previously [24]. PCR amplicons were purified using AMPure XP magnetic purification beads (Beckman Coulter, Inc., Brea, CA, USA) and quantified using the Quant-iT PicoGreen ds DNA Assay Kit (Life Technologies Japan, Ltd., Tokyo, Japan). The pooled amplicons were sequenced using MiSeq (Illumine, Inc., San Diego, CA, USA) according to the manufacturer’s instructions.

### Data analysis

The demultiplexed reads were processed with the DADA2 package [25] in R for quality filtering, denoising, chimera removal, and inference of ASVs. ASVs were taxonomically annotated against the SILVA database (v138.1). Downstream analyses were conducted with the phyloseq package [26]. Differentially abundant taxa between groups were identified using indicator species analysis [27] and linear discriminant analysis effect size (LEfSe) [28], implemented with the indicspecies and microbial packages, respectively. The sequencing data are available at the DDBJ under accession number BioProject: PRJDB40650.

### Statistical analysis

Log-rank (Mantel‒Cox) test, the Gehan‒Breslow‒Wilcoxon test, one-way or two-way ANOVA and Tukey's multiple comparisons test were used. All analyses were performed using Prism (GraphPad Software version 10.4.1; La Jolla, CA, USA).

## Results

### Altered gut microbiota caused by antibiotic administration ameliorates experimental cerebral malaria

**Fig. 1:**
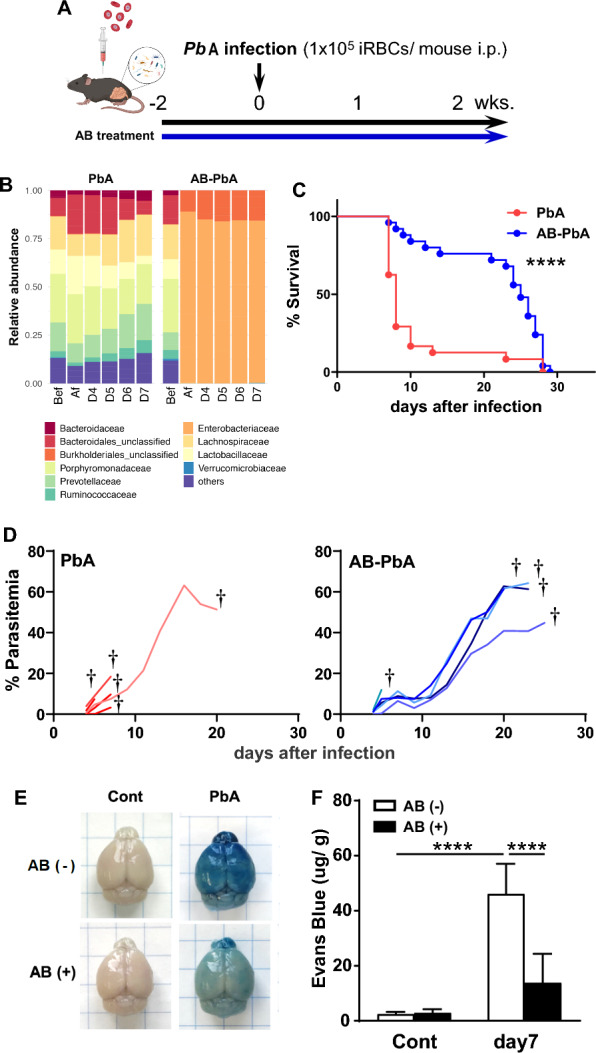
Altered gut microbiota caused by antibiotic administration ameliorates experimental cerebral malaria. **a** C57BL/6 mice were treated with four antibiotics (AB) in the drinking water for 2 weeks before infection and during infection. The mice were infected with PbA on Day 0, and parasitemia was monitored every 2–3 days. **b** Relative abundance of the fecal bacterial family in mice treated with AB and controls at the indicated time points. Survival (**c**) and parasitemia (**d**) of mice treated with AB and controls. Survival is from 25 AB-PbA mice and 24 PbA mice; representative parasitemia values are from 5 AB-PbA and 5 control mice with PbA. Statistical analysis was performed using the Log-rank (Mantel‒Cox) test and the Gehan‒Breslow‒Wilcoxon test for survival. **e**, **f** Mice were injected with Evans blue (EB) dye 7 days after infection. Uninfected mice were used as controls. Representative photographs of the brain (**e**) and amounts of EB per g of brain (**f**) are shown. The data are presented as the means ± SDs from 7 to 10 mice. Statistical analysis was performed using two-way ANOVA and Tukey's multiple comparisons test. ****p < 0.0001

To investigate whether the host intestinal microbiota affects the pathogenesis of ECM, we modified the gut microbiota of B6 mice using drinking water supplemented with four antibiotics (4AB), ampicillin (Amp), neomycin (Neo), metronidazole (Met), and vancomycin (Van) for 2 weeks, after which the mice were infected with PbA (Fig. 1a). Compared with the control mice, the 4AB-treated B6 mice exhibited significant changes in the composition of the microbiota after treatment, but the composition remained stable during PbA infection (Fig. 1b). Notably, approximately 80% of 4AB-treated B6 mice with PbA infection avoided ECM and died with high parasitemia by 4 weeks after infection (Fig. 1c, d). To evaluate the development of ECM, Evans blue (EB) dye was intravenously injected into mice, and the leakage of EB dye was significantly reduced in 4AB-treated mice infected with PbA, suggesting that the ECM was ameliorated (Fig. 1e, f).

### Cancellation of lymphocyte infiltration into the brains of 4AB-PbA mice

**Fig. 2:**
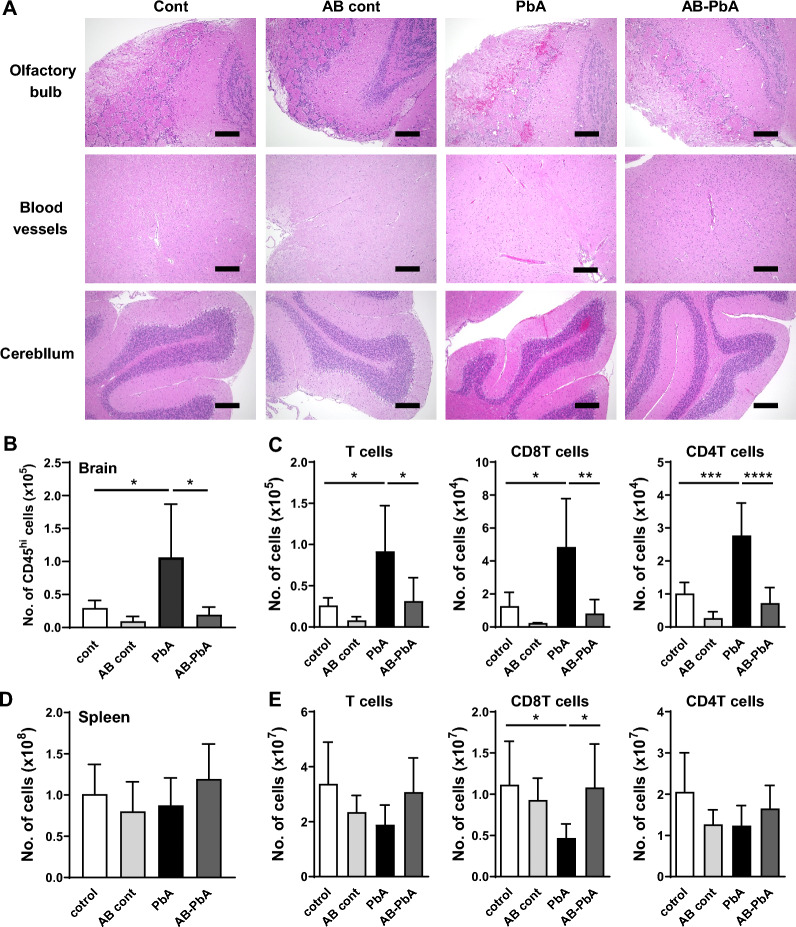
Prevention of lymphocyte infiltration into the brains of 4AB-PbA mice. **a** Brains from AB-treated and control mice that were uninfected or 7 days after infection were stained with H&E. Scale bars = 200 μm. **b**–**e** Phenotypic characterization of lymphocytes by flow cytometric analysis in AB-treated mice and controls. Mononuclear cells per brain or spleen of AB-treated and control mice were harvested before infection and 7 days after infection. The data are the means ± SDs. The numbers of CD45^hi^ white blood cells (**b**) and T-cell subsets (CD3T, CD8T and CD4T cells) (**c**) in the brain. The numbers of lymphocytes (**d**) and T-cell subsets (CD3T, CD8T and CD4T cells) (**e**) in the spleen. The data are presented as the means ± SDs from 3 to 10 mice per group. Statistical analysis was performed using Ordinary one-way ANOVA and Tukey's multiple comparisons test. *p < 0.05, **p < 0.01, ***p < 0.001, ****p < 0.0001

Next, the brain pathology of 4AB-treated mice was examined 7 days after infection, at which time the ECM was ameliorated. Hemorrhage, adhesion, and vascular occlusion of red blood cells were observed in the olfactory bulb (100.0 ± 0.0%), cerebellum (100.0 ± 0.0%), cerebral parenchyma (95.0 ± 15.8%), and cerebral blood vessels (95.0 ± 15.8%) in B6 mice on Day 7 of PbA infection, which caused neurological symptoms (Fig. 2a, Table 1). On the other hand, 40% of 4AB-treated B6 mice were free from hemorrhage, similar to uninfected mice, although other mice had hemorrhage, adhesion, and vascular occlusion of red blood cells in the olfactory bulb (55.0 ± 0.0%), cerebellum (45.0 ± 43.8%), cerebral parenchyma (25.0 ± 42.2%), and cerebral blood vessels (35.0 ± 41.2%). These findings indicate that the cerebral pathology in 4AB-treated B6 mice was mild. To clarify the magnitude of leukocyte infiltration into the brain, which results in inflammation, leukocytes were isolated from the brain and detected by flow cytometry. CD45^hi^ white blood cell infiltration was observed in mice with PbA infection (Fig. 2b). On Day 7 of PbA infection, however, the number of infiltrating leukocytes in 4AB-treated B6 mice was significantly lower than that in PbA-infected mice (Fig. 2b). Most of the lymphocytes that infiltrated the brains of PbA-infected mice were T cells, including CD8T cells and CD4T cells, and the number of CD8T cells decreased in the spleen (Fig. 2c, d). However, the increase in T cells associated with brain pathogenesis was reversed in 4AB-treated B6 mice. These findings suggest that cerebral symptoms were reduced in mice in which the bacterial flora was altered by the administration of antibiotics, as erythrocyte adhesion to the brain and leukocyte infiltration improved.

**Table 1:** Histological analyses of the brains of mice infected with PbA

	Olfactory bulb (%)	Cerebral parenchyma (%)	Cerebellum (%)	Cerebral blood vessels (%)
Cont (n = 8)	0.0 ± 0.0	0.0 ± 0.0	0.0 ± 0.0	25.0 ± 37.8
AB cont (n = 8)	31.3 ± 45.8	6.3 ± 17.7	0.0 ± 0.0	18.8 ± 37.2
PbA (n = 10)	100.0 ± 0.0	95.0 ± 15.8	100.0 ± 0.0	95.0 ± 15.8
AB-PbA (n = 10)	55.0 ± 49.7	25.0 ± 42.5	45.0 ± 43.8	35.0 ± 41.2

Brains from mice that were uninfected or 7 days after infection were stained with H&E. The presence of red blood cell hemorrhage and vascular occlusion in the olfactory bulb, cerebellum, cerebral parenchyma, and cerebral blood vessels were examined in two fields of the right and left hemispheres of each mouse. The data are presented as the means ± SDs from 8 to 10 mice

### Some species of microbiota correlate with ECM development

**Fig. 3:**
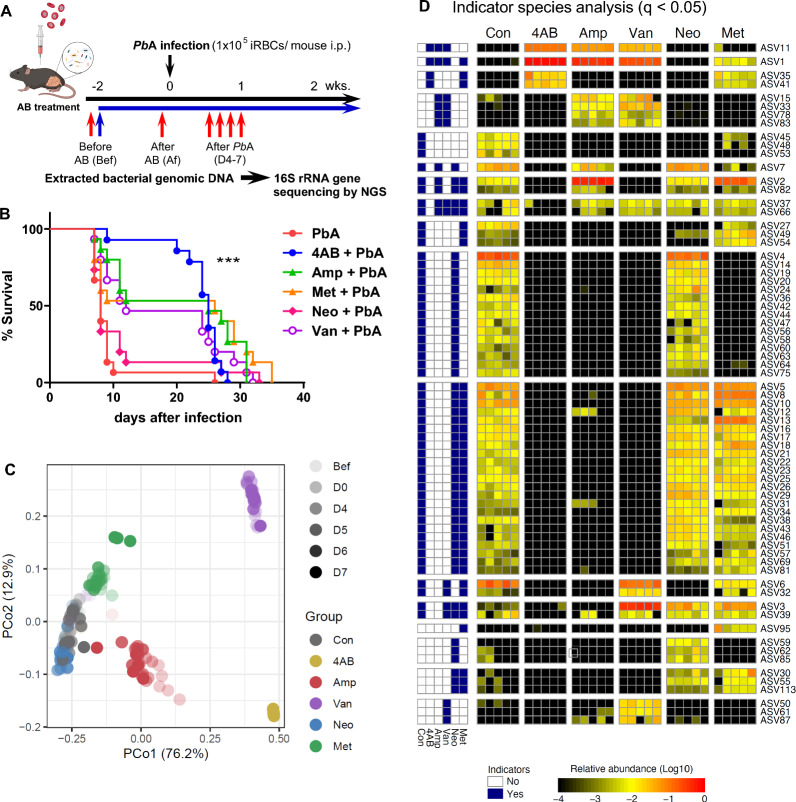
Some species of microbiota correlate with ECM development. **a** C57BL/6 mice were treated with 4AB, ampicillin (Amp), neomycin (Neo), metronidazole (Met) or vancomycin (Van), in drinking water from 2 weeks before infection and during infection. Stool samples were collected from 4AB-treated, Amp-treated, Neo-treated, Met-treated and Van-treated mice before treatment (Bf) and after treatment (Af) and at 4–7 days after infection. **b** Survival curve after infection. Survival data are obtained from 20 mice per group for 4 individual experiments. **c** Principal coordinate analyses based on the weighted UniFrac distance of the fecal microbiota from 4AB-treated, Amp-treated, Neo-treated, Met-treated and Van-treated mice before treatment (Bf) and after treatment (Af) and at 4–7 days after infection. Each symbol represents an individual mouse at different time periods and is distinguished by different colors as indicated. **d** Indicator species analysis at time points Af. Each row represents an individual bacterial ASV, and each column represents an individual mouse. The color scale represents the ASV abundance. Statistical analysis was performed using the Log-rank (Mantel‒Cox) test and the Gehan‒Breslow‒Wilcoxon test for survival. ***p < 0.001

Because milder ECM symptoms and changes in the intestinal microbiota were observed following the administration of antibiotics, we attempted to identify the bacteria associated with ECM development during PbA infection by administering a single antibiotic (Fig. 3a). Stool samples were collected from 4AB-treated, Amp-treated, Neo-treated, Met-treated and Van-treated mice before treatment (Bf) and after treatment (Af) and at 4–7 days after infection. A single administration of ampicillin, metronidazole, or vancomycin-treated B6 mice infected with PbA reduced CM survival to 50%. Moreover, the survival curve of neomycin-treated mice was similar to that of untreated mice (Fig. 3b). These results show that the alleviation of cerebral symptoms occurred only when 4AB was administered.

Bacterial 16S rRNA gene sequences were assigned to species-level ASVs. The results of principal coordinate analyses bacterial composition and weighted UniFrac distance determination revealed that the intestinal microbiota in 4AB-treated mice changed markedly after treatment and was significantly different from that in the other groups (Fig. 3c; Additional file: Figs. S1a-b). Indicator species analysis, a method for comparing microbiota compositions, revealed that ASV1 (a common denominator of 4AB, Amp, Met and Van), ASV11 (a common denominator of 4AB, Amp and Van) and ASV35 and ASV41 (a common denominator of 4AB and Met) were enriched before infection to Day 7 (Fig. 3d; Additional file: Fig. S2). Moreover, ASV37 and ASV66 expression decreased in only 4AB-treated mice before infection (Fig. 3d).

To identify bacteria associated with death within 2 weeks by ECM, LEfSe revealed that 49 species of bacteria were related to death due to ECM, and 6 species of bacteria were related to survival (Fig. S2a). After PbA infection, 46 species of bacteria were associated with death due to ECM, and 7 species of bacteria were associated with survival. ASV changes were not observed during infection. 14 species of bacteria for ECM and 6 species of bacteria for survival were common both before and after infection. ASV37 and ASV66 decreased in only in 4AB-treated mice were associated with death due to ECM from Day 4 to Day 7 (Fig. S2b).

### Influence of gut microorganisms on ECM development

**Fig. 4:**
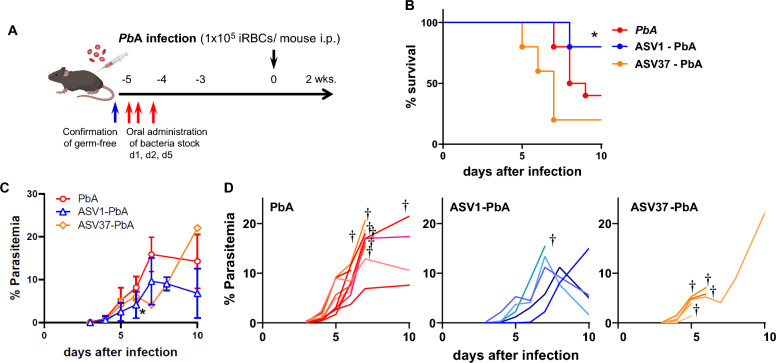
Gut bacteria influence ECM development. **a** ASV1 or ASV37 bacteria was orally administered 3 times to germ-free C57BL/6 mice, and colonized mice were infected with PbA on Day 0. **b** Survival curve after infection in ASV1- and ASV37-colonized gnotobiotic- and control-specific pathogen-free mice. Averages of parasitemia (**c**) and individual data of parasitemia (**d**) after infection in 5 ASV1-colonized mice, 5 ASV37-colonized mice and 10 specific pathogen-free mice. Statistical analysis was performed using one-way ANOVA and Tukey's multiple comparisons test and the Gehan-Breslow-Wilcoxon test for survival. *p < 0.05

To clarify the influence of a single bacterium on the development of ECM and to exclude the effects of antibiotics on ECM, we isolated viable bacteria corresponding ASV1, the bacterium showing the greatest increase in the 4AB group, and ASV37, a bacterium whose abundance decreased only in the 4AB group. Both isolates from fresh stool samples showed a 100% match in the V4 region of the 16S rRNA gene. Germ-free mice were then monocolonized with ASV1 or ASV37, followed by a gnotobiotic experiment and PbA infection (Fig. 4a). There was no difference in the survival rate between PbA-infected B6[SPF] and B6[GF] mice (Fig. S3a). ASV1-colonized gnotobiotic (GB) mice showed delayed ECM and mortality, indicating low parasitemia throughout the infection (Fig. 4c, d), similar to the results of the fecal microbiota transplant from AB-treated mice to GF mice (Fig. S3b). On the other hand, ASV37-colonized GB mice showed neurological symptoms beginning on Day 4, earlier than controls, and 80% of the mice died before Day 7. This finding suggests that intestinal bacteria, not antibiotics, are involved in ECM pathogenesis.

## Discussion

The influence of the gut microbiota on malaria infection, which involves changes in the gut microbiota, immune dynamics, and pathological conditions, has been frequently reported in malaria patients and murine models [11–17, 29–33]. Here, the results of the present study revealed that the altered gut microbiota prevented ECM-induced lethality in 80% of AB-treated mice (Fig. 1c). Multiple bacterial species were identified and predicted to affect the development of ECM, including ASV1 and ASV37. Moreover, ASV1- and ASV37-colonized GF mice exhibited some changes in PbA infection and ECM pathogenesis (Figs. 3d, 4a–d and Figs. S2a-b).

The parasite burden of murine malaria infection in germ-free mice did not markedly differ from that in SPF mice [30, 32, 34], although differences in survival were observed between *P. berghei* K173-infected GF and SPF mice [32]. Consistent with this finding, PbA-infected GF mice did not differ from SPF mice, and the gut microbiota did not seem to be involved in the pathology of PbA infection. However, BBB permeability was higher in GF mice, and administration of bacteria to GF mice resulted in closely aligned BBB tight junctions [35]. Therefore, in GF mice, the absence of a microbiota seems to increase BBB permeability during ECM. In SPF mice, the tight junctions of the BBB were maintained by the presence of the gut microbiota, but BBB permeability increased because of changes in the gut microbiota caused by PbA infection in addition to immune responses to PbA.

Notably, Lactobacillaceae, as shown in ASV37, seemed to be associated with ECM-related lethality, and Enterobacteriaceae, as shown in ASV1, seemed to function as an ECM-resistance factor in this experiment, although the abundance of Lactobacillaceae decreased and that of Enterobacteriaceae increased following PbA infection in B6 mice, as previously reported [11]. *Lactobacillus reuteri* (ASV1) or *Klebsiella pneumoniae* (ASV37) gnotobiotic experiments revealed a trend toward differences in survival rates and parasitemia depending on colonized bacteria, suggesting that these bacteria are associated with ECM pathogenesis. These results may reconsider the interpretation that Lactobacillaceae, which decreases in abundance following infection, is beneficial to host defense, whereas an increase in Enterobacteriaceae abundance is detrimental.

With respect to Lactobacillaceae, including ASV37, and Muribaculaceae identified in the LEfSe analysis, as shown in Figs. S2, the majority of biomarkers associated with ECM-related lethality before infection belonged to Lachnospiraceae (18/49 species) and Muribaculaceae (11/49 species). After infection, the majority shifted to Muribaculaceae (21/46 species) and Lactobacillaceae (5/46 species), indicating that the candidate bacteria changed following infection. LEfSe analyses in the literature have revealed the involvement of Lactobacillaceae and Muribaculaceae in murine malaria [31, 32, 33, 36]. Analysis of the lung microbiota in PbA-infected mice revealed that Lactobacillaceae and *Ligilactobacillus* may serve as biomarkers for high parasitemia, based on comparisons among high parasitemia, uninfected, and schizont membrane-associated cytoadherence protein groups [32]. Lactobacillaceae (4/11 species) was also identified as a biomarker for PbA infection on Day 4 in a comparison between PbA-infected and uninfected mice [33]. During *P. yoelii* 17XL (PyL) infection, Lactobacillaceae (4/5 species) could be a key biomarker for PyL infection on Day 2, and Muribaculaceae (2/8 species) was identified as a biomarker for PyL infection on Day 5 based on comparisons among Day 2, Day 5 and uninfected mice in a LEfSe analysis [29]. The effect of intestinal bacteria on parasitemia during PyNL has been demonstrated by the reduction in parasites in susceptible B6 (NCI/Har) mice treated with *Lactobacillus* and *Bifidobacterium* [16].

*L. reuteri*, a probiotic bacterium of the genus *Lactobacillus*, and its metabolites have multiple effects on the host via immunomodulation, the intestinal barrier, protection from pathobionts, translocation to extraintestinal sites and resistance to intestinal pH and bile salts [37–39]. Although the functions of *L. reuteri* in terms of intestinal barrier integrity and protection from pathobionts are expected to have positive effects on ECM, our results from indicator species analysis and gnotobiotic experiments did not reveal these positive effects. In PyNL infection, *L. reuteri* has been reported as one of the bacterium that is significantly abundant in mice susceptible to hyperparasitemia [17]. *L. reuteri* has immunomodulatory effects, and the AhR ligand indole-3-aldehyde produced by *L. reuteri* increases the number of IFN-γ-secreting CD8 T cells and leads to the development of autoimmune hepatitis-like disease [40]. Furthermore, in experimental autoimmune encephalomyelitis (EAE), an animal model of multiple sclerosis, mice colonized with *L. reuteri* with a peptide that may mimic MOG and *Erysipelotrichaceae* showed more severe symptoms of EAE than germ-free or mono-colonized mice, suggesting that gut microbes, including *L. reuteri*, are involved in the onset and severity of multiple sclerosis [41, 42]. The role of *L. reuteri* in IFN-γ secretion during malaria infection remains unknown, but CD8 T cells play a crucial role in the pathogenesis of ECM, suggesting that *L. reuteri* may be involved in this process through the induction of these cells. The function of *L. reuteri* varies depending on the strain [37, 43]; thus, these results may also be due to differences in the strain. However, further detailed studies with larger sample sizes are needed to evaluate the effects of *L. reuteri* on ECM.

Enterobacteriaceae, which contain many pathobionts, have been frequently detected in malaria studies [10, 13, 17], but their roles also remain unclear. Galα1-3Galβ1-4GlcNAc-R (α-gal) glycan, which is expressed in enterobacteria, including *Klebsiella* spp., *Serratia* spp., and *Escherichia coli*, is also expressed on the surface of malaria parasites, suggesting that anti-α-Gal IgM antibodies induce a protective immune response against malaria transmission [10]. Transfer of *E. coli* to GF mice reduces parasitemia, suggesting that *E. coli* contributes to the reduction in parasitemia during PyNL infection [17]. Escherichia-Shigella has been shown to be a phylotype biomarker for resistance to PyNL infection by LefSe [16]. *E. coli* and *K. pneumoniae* are increased in severe malaria anemia patients, indicating that the composition of the intestinal microbiota is associated with the severity of falciparum malaria [13]. In our study, the abundance of *Klebsiella* was high in AB-treated mice that avoided ECM, and the inoculation of *Klebsiella* and FMT, including *Klebsiella,* into germ-free mice may have a protective effect against cerebral symptoms. However, the trend was not significant, suggesting that controlling pathogenicity with a single bacterium alone may be difficult. In this study, we compared the bacterial flora in six groups, including those treated with a single antibiotic, and identified more bacteria potentially associated with ECM-related lethality than with survival. These results may be because many bacteria are affected by antibiotics, leaving only a few residual taxa. Although this study used six groups and included single antibiotic treatments, increasing the number of groups with two- or three-antibiotic combinations may help narrow down the bacterial taxa more precisely associated with cerebral symptoms. Further experiments using this system will enable us to identify the bacteria that contribute to disease exacerbation and those that are involved in amelioration, as well as to elucidate the mechanisms through which they influence ECM.

## Conclusions

This study provides compelling evidence that the gut microbiota is a key determinant of the development and severity of ECM. This finding shows that not only the presence or absence of certain bacteria but also the overall composition of the gut microbiome can profoundly affect disease outcomes. Changes in the intestinal environment caused by PbA infection, including gut dysbiosis and intestinal pathology, may be not simply a disadvantage to the host but may also act as a natural defense against malaria. These insights open new avenues for research into microbiota-based therapies for malaria and other diseases involving neuroinflammation and immune dysregulation.

## Supplementary Information

Additional file 1: Figure 1. Dynamic changes in stool bacterial components from 4AB-treated, Amp-treated, Neo-treated, Met-treated and Van-treated mice before and after infection with *Plasmodium berghei *ANKA. Related to Fig 3. (a) Relative abundance of bacterial genera in the feces of control (Con) and 4AB-treated, Amp-treated, Neo-treated, Met-treated and Van-treated mice. Each bar represents the average result for 5 mice at the indicated time points. (b) Weighted UniFrac distance of the fecal microbiota from the Control (Con) and 4AB-treated, Amp-treated, Neo-treated, Met-treated and Van-treated mice. Each boxplot represents the results from 5 mice at the indicated time points. Statistical analysis was performed using Dunnett’s test. *p < 0.05, **p < 0.01, ***p < 0.001 compared with uninfected mice. Figure 2. Taxa prediction using LEfSe provides information about the death resulting from experimental cerebral malaria in mice. Related to Fig 3. LEfSe analysis revealed a family of microbes whose abundance significantly differed between ECM-related death (Y) and survival (N) before infection (a) and between Days 4 and 7 after infection (b). The linear discriminant analysis (LDA) score at the log10 scale is indicated at the bottom. The greater the LDA score is, the more significant the functional biomarker is in the comparison. Figure 3. Transplanted fecal microbiota from 4AB-treated mice into gnotobiotic mice prevented ECM. Related to Fig 4. (a) Differences in survival between 5 B6[SPF] and 5 B6[GF] mice infected with PbA. (b) The fecal microbiota from 4AB-treated bacteria was orally administered 3 times to germ-free B6 mice, and colonized mice were infected with PbA on Day 0. Differences in survival between 5 B6[SPF] and 5 B6[GB] mice infected with PbA.

## Data Availability

All the data generated or analyzed during the current study are available from the corresponding author upon reasonable request.

## Abbreviations

PbA: *Plasmodium berghei* ANKA
ECM: Experimental cerebral malaria
BBB: Blood–brain barrier
B6: C57BL/6N mice
SPF: Specific-pathogen-free
GF: Germ-free
GB: Gnotobiotic
4AB: Four antibiotics
ASVs: Amplicon sequence variants
