# Supplementary figures and images for: Gut microbiota bidirectionally influences protection and severity in cerebral malaria in mice
Source: Trop Med Health. 2026 Apr 16;54:78. doi: 10.1186/s41182-026-00947-1 (PMC13151302; doi:10.1186/s41182-026-00947-1)

**Fig. S1**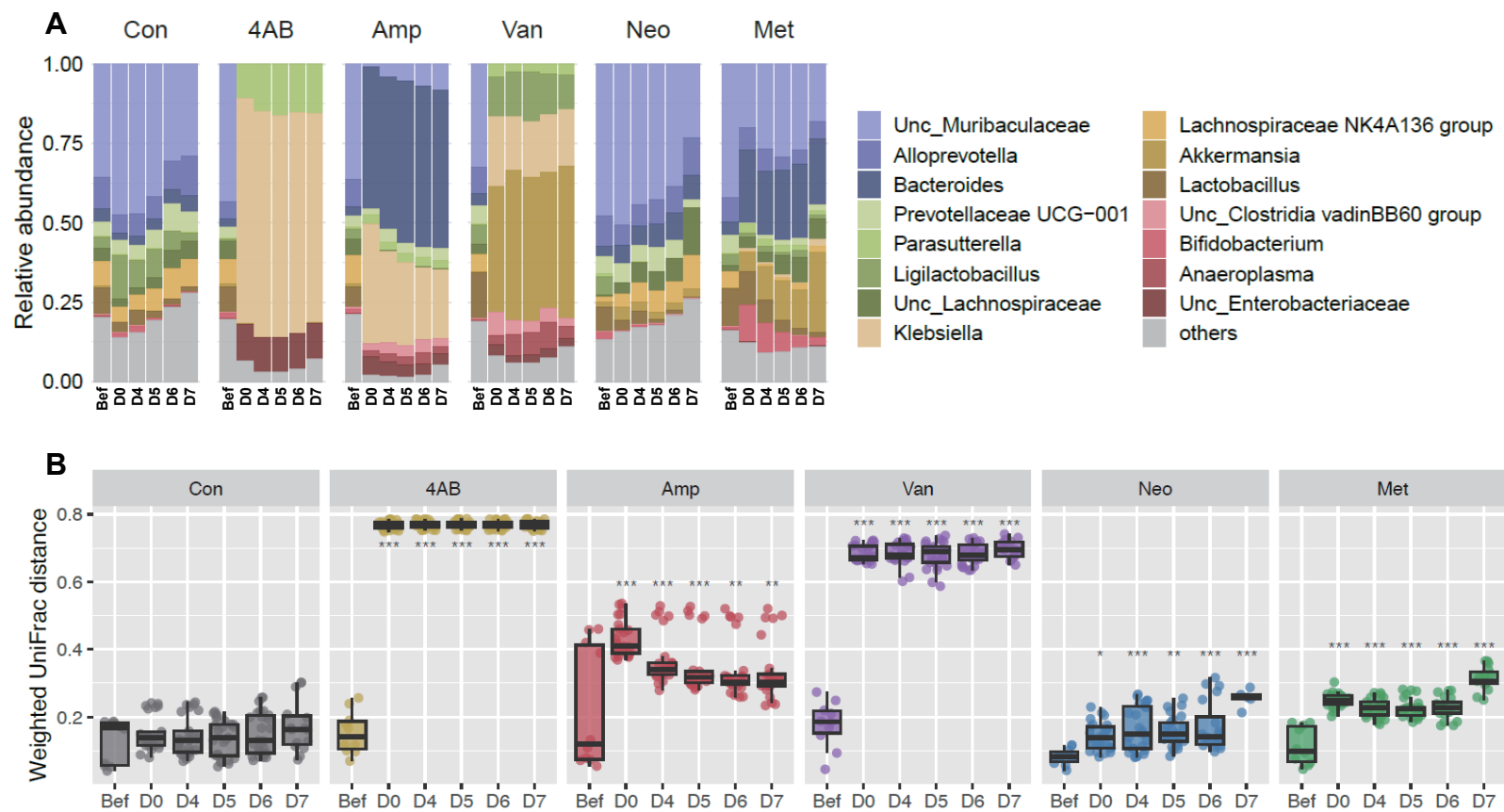

Fig. S2

A day0

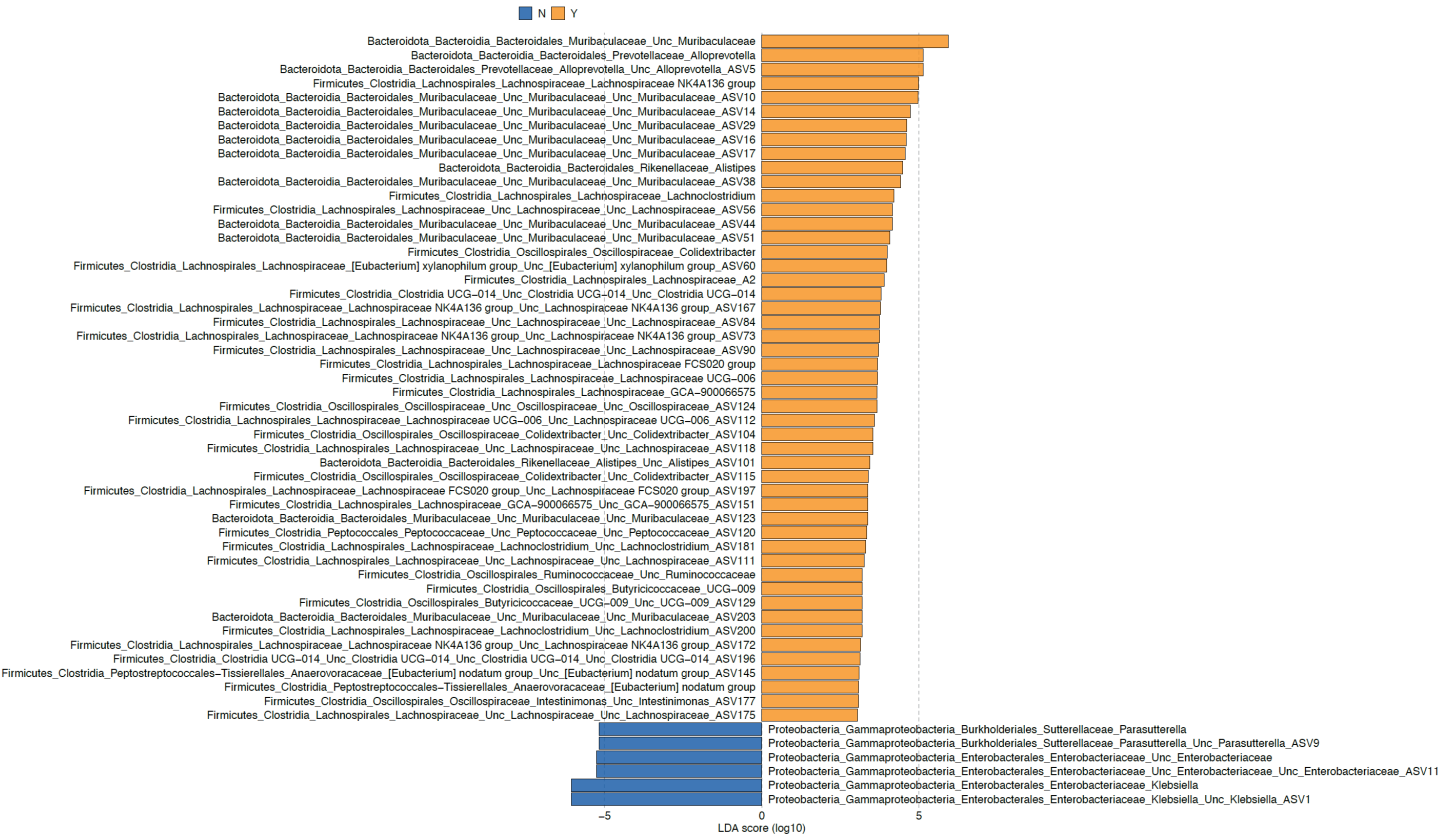

B day4 - day7

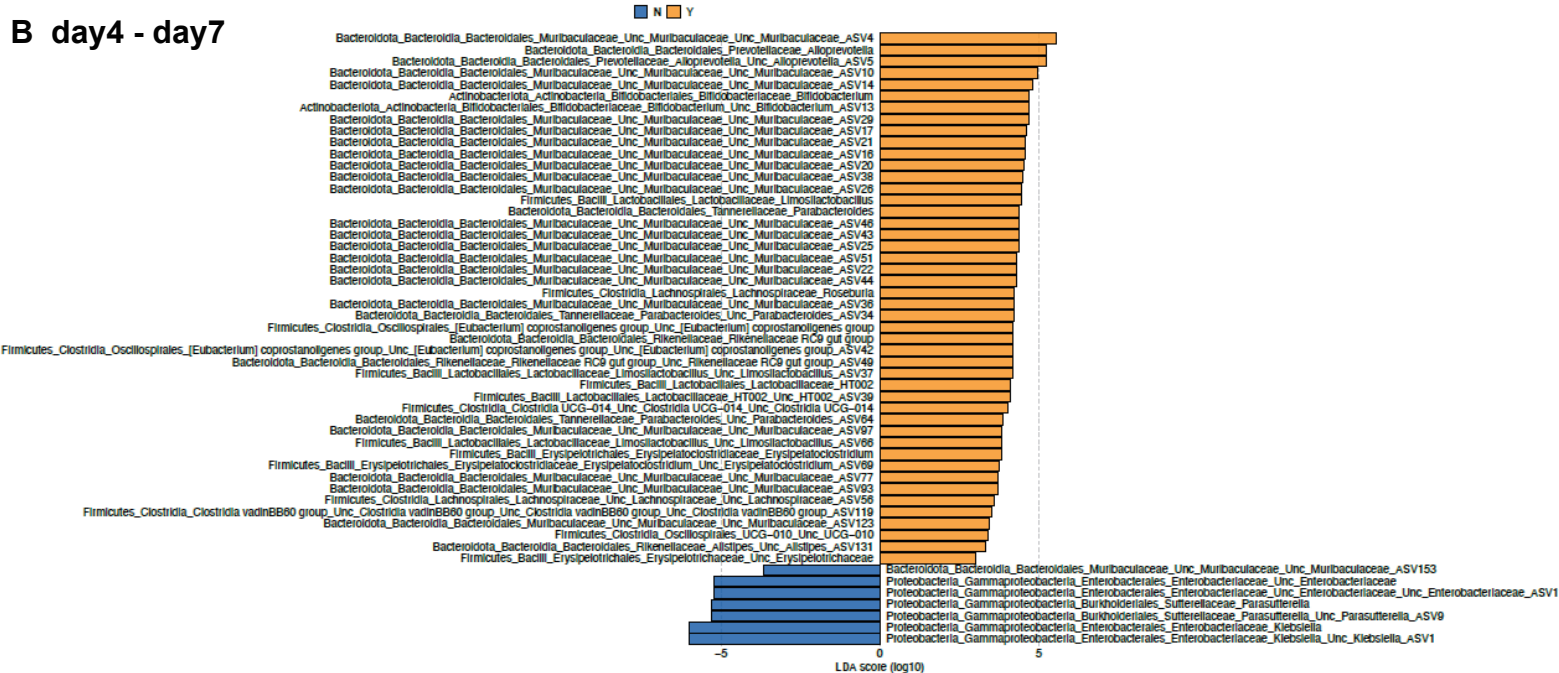

Fig. S3

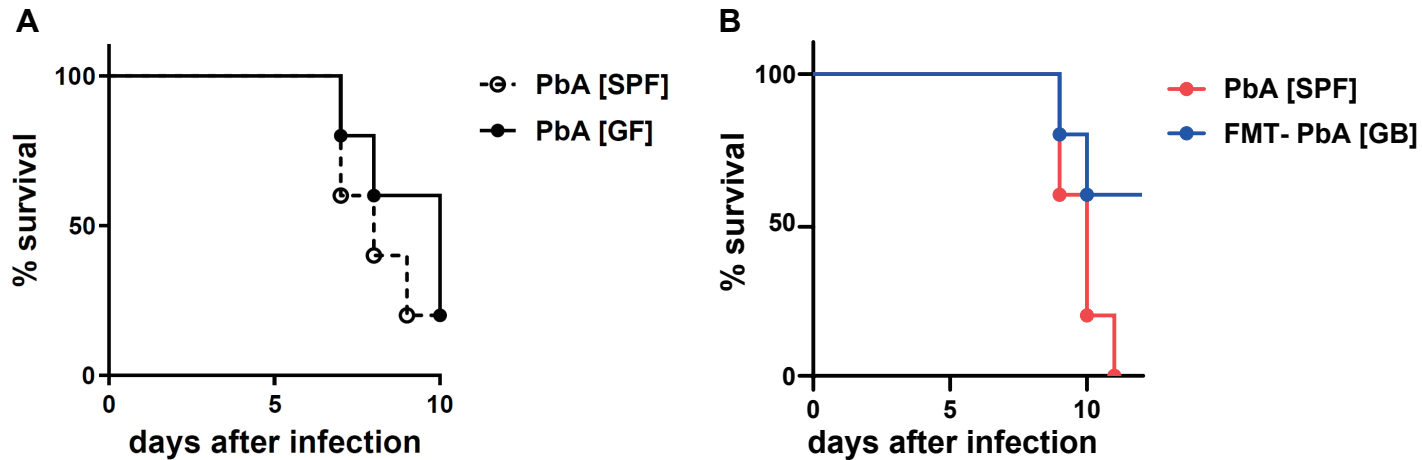

Supplement: Supplementary file 1 — Additional file 1: Figure 1. Dynamic changes in stool bacterial components from 4AB-treated, Amp-treated, Neo-treated, Met-treated and Van-treated mice before and after infection with Plasmodium berghei ANKA. Related to Fig 3. (a) Relative abundance of bacterial genera in the feces of control (Con) and 4AB-treated, Amp-treated, Neo-treated, Met-treated and Van-treated mice. Each bar represents the average result for 5 mice at the indicated time points. (b) Weighted UniFrac distance of the fecal microbiota from the Control (Con) and 4AB-treated, Amp-treated, Neo-treated, Met-treated and Van-treated mice. Each boxplot represents the results from 5 mice at the indicated time points. Statistical analysis was performed using Dunnett’s test. *p < 0.05, **p < 0.01, ***p < 0.001 compared with uninfected mice. Figure 2. Taxa prediction using LEfSe provides information about the death resulting from experimental cerebral malaria in mice. Related to Fig 3. LEfSe analysis revealed a family of microbes whose abundance significantly differed between ECM-related death (Y) and survival (N) before infection (a) and between Days 4 and 7 after infection (b). The linear discriminant analysis (LDA) score at the log10 scale is indicated at the bottom. The greater the LDA score is, the more significant the functional biomarker is in the comparison. Figure 3. Transplanted fecal microbiota from 4AB-treated mice into gnotobiotic mice prevented ECM. Related to Fig 4. (a) Differences in survival between 5 B6[SPF] and 5 B6[GF] mice infected with PbA. (b) The fecal microbiota from 4AB-treated bacteria was orally administered 3 times to germ-free B6 mice, and colonized mice were infected with PbA on Day 0. Differences in survival between 5 B6[SPF] and 5 B6[GB] mice infected with PbA. [file 41182_2026_947_MOESM1_ESM.pdf]
